# Cannabis Withdrawal and Psychiatric Intensive Care

**DOI:** 10.1001/jamapsychiatry.2025.1216

**Published:** 2025-06-11

**Authors:** Aliyah Malik, Hitesh Shetty, Dominic Oliver, Thomas J. Reilly, Marta Di Forti, Philip McGuire, Edward Chesney

**Affiliations:** 1 Department of Addictions, Institute of Psychiatry, Psychology & Neuroscience, King’s College London, United Kingdom; 2 South London and Maudsley NHS Foundation Trust, London, United Kingdom; 3 Department of Psychiatry, University of Oxford, Oxford, United Kingdom; 4 NIHR Oxford Health Biomedical Research Centre, Oxford, United Kingdom; 5 Social, Genetic & Developmental Psychiatry Centre, Institute of Psychiatry, Psychology & Neuroscience, King’s College London, United Kingdom

## Abstract

**Importance:**

Cannabis use is common in people with severe mental illness and its adverse effects on outcomes are well established. However, adverse outcomes may also result from cannabis withdrawal syndrome (CWS). CWS includes symptoms such as agitation, irritability, and aggression, and typically peaks after 3 to 5 days of abstinence.

**Objective:**

To assess whether cannabis use prior to admission is associated with an increase in the risk of transfer to a psychiatric intensive care unit (PICU) during the cannabis withdrawal risk period.

**Design, Setting, and Participants:**

This retrospective cohort study used clinical data from a secondary mental health care database and took place at 4 psychiatric hospitals in London, United Kingdom, between January 2008 and December 2023. Patients included adults admitted to general psychiatric wards and PICUs. Data were analyzed from June 2023 to February 2025.

**Exposure:**

Cannabis use was determined from clinical records, using natural language processing and manual review.

**Main Outcomes and Measures:**

The primary outcome was transfer from a general ward to PICU during the cannabis withdrawal risk period (3 to 5 days after presentation to the hospital). Secondary outcomes included admission to PICU at any time point. Outcomes were analyzed according to cannabis use status with multivariable models, which adjusted for age, gender, ethnicity, diagnosis, tobacco use, stimulant use, comorbid alcohol or substance use disorder, and admission year.

**Results:**

There were 52 088 hospital admissions identified, of which 4691 involved admission to a PICU (9.0%). Cannabis users were more likely to be admitted to a PICU than nonusers (adjusted odds ratio [aOR], 1.44; 95% CI, 1.33-1.55; *P* < .001). There were 1236 admissions where the patient was transferred to PICU after initial admission to a general ward (mean [SD] age, 33.4 [10.4] years; 810 male [66%] and 426 female [34%]). At 3 to 5 days postpresentation (the risk period for cannabis withdrawal), transfer from a general ward to a PICU was more common in cannabis users (31.0%) than nonusers (24.2%) (aOR, 1.36; 95% CI, 1.01-1.81; *P* = .04). The association was particularly evident in women (aOR, 2.03; 95% CI, 1.22-3.39; *P* = .007) and in those older than 35 years (aOR, 2.53; 95%CI: 1.52-4.21; *P* < .001).

**Conclusions and Relevance:**

People with severe mental illness who are cannabis users may develop cannabis withdrawal syndrome shortly after hospital admission, and this can exacerbate their mental state.

## Key Points

**Question:**

Does cannabis withdrawal increase the risk of admission to psychiatric intensive care?

**Findings:**

In psychiatric inpatients, cannabis use prior to admission was associated with an increased risk of admission to intensive care. This risk was particularly elevated 3 to 5 days after presentation to hospital, a period associated with the peak severity of cannabis withdrawal syndrome.

**Meaning:**

In people with psychiatric disorders, cannabis withdrawal may occur shortly after hospital admission and exacerbate their mental state.

## Teaser

This study attempts to assess whether cannabis use prior to admission is associated with an increase in the risk of transfer to a psychiatric intensive care unit during the cannabis withdrawal risk period.

## Introduction

Cannabis use in people with severe mental illness can exacerbate psychotic symptoms and is associated with an increased risk of violence.^1,2^ A study in London, United Kingdom, found that recent cannabis users had 5 times higher odds of violence during emergency psychiatric assessments.^3^ Those with cannabis dependence may also experience adverse outcomes as a result of cannabis withdrawal syndrome (CWS).^4,5^ CWS has been described following the abrupt cessation of cannabis use in heavy users. It typically begins within 24 to 48 hours and peaks between 2 to 6 days.^4^ The symptoms include anxiety, irritability, anger, disturbed sleep, restlessness, depressed mood, loss of appetite, headache, and sweating.

There are no recommended treatments for CWS in clinical practice. Therefore, people with cannabis dependence are at risk of developing CWS when they are admitted to hospital. The objective of the present study was to investigate the association between cannabis use prior to hospital admission and the subsequent need for psychiatric intensive care unit (PICU), an index of deteriorating mental state and increased risk of violence and aggression.^6^ We focused on the period 3 to 5 days after presentation to hospital, when CWS is most likely to be evident. This hypothesis was preregistered.^7^

## Methods

Data were obtained from the South London and Maudsley NHS Foundation Trust using the Clinical Record Interactive Search system.^8^ The Trust has 4 psychiatric hospitals and 4 PICUs. Ethical approval was provided by Oxfordshire REC C (23/SC/0257). As the data are anonymized, the requirement for consent was waived in accordance with the European Data Protection Directive. All patients have the option to remove their data from the database. The observation period was defined as January 1, 2008, to December 31, 2023, excluding March 26, 2020, to April 26, 2022, due to the COVID-19 pandemic. Any adult patient who was admitted to the general psychiatric ward or PICU during this period was included in the study. The study followed the Reporting of Studies Conducted Using Observational Routinely-Collected Data (RECORD) guidelines.^17^

The primary outcome was the risk of transfer to PICU during the cannabis withdrawal risk period (days 3 to 5 after presentation). It was defined as the time from initial presentation to an emergency department or other assessment unit, until referral or admission to PICU. Any participant who was initially admitted to a general psychiatric ward and then transferred to PICU within 14 days was included in the analysis.

Each participant’s/admission’s case notes were reviewed manually to determine cannabis use status prior to hospital admission and was classified into 1 of 5 groups: current user, past user (abstinent for more than 4 weeks), recently abstinent user (between 2 days and 4 weeks), never/minimal user, and not determined. Interrater reliability (Cohen κ) was 0.88 (eMethods 1 Supplement 1). Past users and never/minimal users were combined into a single nonuser group.

The secondary outcome was the risk of admission or transfer to PICU at any time point. This analysis included all participants and used a natural language processing (NLP) application to determine cannabis use status.

We used validated NLP applications to identify cannabis, tobacco, and stimulant (cocaine, crack cocaine, amphetamines, and MDMA) use from case notes (+/− 1 week from admission). Substance use data are available in health records, as they are a standard part of psychiatric assessment for inpatients. These applications have a high precision (87% to 97%) for detecting substance use in electronic health records.^9^

### Statistical Analysis

Primary and secondary outcomes were analyzed with multivariable logistic regression models both unadjusted and adjusted for covariates (age, gender, ethnicity [self-reported], primary diagnosis, comorbid diagnosis of an alcohol or substance use disorder [excluding cannabis use disorder], tobacco use, stimulant use [cocaine/amphetamine/MDMA], and year of admission), with α set at *P* < .05. All analyses were completed using Stata version 18.0 (Stata Corp).

## Results

### Cannabis Use and Admission or Transfer to PICU at Any Time Point

A total of 52 088 hospital admissions were identified during the observation period (eFigure 1 and eTable 1 in Supplement 1). A greater proportion of cannabis users (3244 of 24 579 [13.2%]) than nonusers (1447 of 27 509 [5.3%]) were admitted to PICU at any time point during their admission (odds ratio [OR], 2.74; 95% CI, 2.57-2.92; *P* < .001; adjusted OR [aOR], 1.44; 95% CI, 1.33-1.55; *P* < .001; eTable 2 in Supplement 1).

### Transfer to PICU During the Cannabis Withdrawal Risk Period

There were 1236 admissions where the patient was first admitted to a general ward before being transferred to a PICU within 14 days (eFigure 1 in Supplement 1; Table 1). The sample included 636 current cannabis users (51.5%) and 600 nonusers (48.5%). The latter comprised 228 past users (18.4%) and 372 never/minimal users (30.1%). Most of these cases had a primary diagnosis of a psychotic (56.6%) or an affective disorder (28.5%). Only 4.4% had a primary substance use disorder.

**Table 1.** Demographic and Clinical Characteristics of the Primary Analysis Population (n = 1236 Admissions)

Characteristic	No. (%)			
	All admissions	Current users	Nonusers	*P* value
No.	1236 (100.0)	636 (51.5)	600 (48.5)	NA
Age, y, mean (SD)	33.4 (10.4)	31.1 (9.4)	35.8 (10.9)	<.001
Gender				
Male	810 (65.5)	488 (76.7)	322 (53.7)	<.001
Female	426 (34.5)	148 (23.3)	278 (46.3)	
Ethnicity^a^				
Black	746 (60.4)	399 (62.7)	347 (57.8)	.04
White	308 (24.9)	139 (21.9)	169 (28.2)	
Other^b^	182 (14.7)	98 (15.4)	84 (14.0)	
Primary diagnosis				
Substance use disorder (F1x^c^)	54 (4.4)	43 (6.8)	11 (1.8)	<.001
Psychotic disorder (F2x^c^)	700 (56.6)	387 (60.8)	313 (52.2)	
Affective disorder (F3x^c^)	352 (28.5)	144 (22.6)	208 (34.7)	
Other	130 (10.5)	62 (9.7)	68 (11.3)	
Cannabis use				
Current user	636 (51.5)	636 (100.0)	NA	NA
Past user	228 (18.4)	NA	228 (38.0)	
Never user	372 (30.1)	NA	372 (62.0)	
Tobacco use				
Yes	845 (68.4)	514 (80.8)	331 (55.2)	<.001
No	391 (31.6)	122 (19.2)	269 (44.8)	
Stimulant use				
Yes	479 (38.8)	340 (53.5)	139 (23.2)	<.001
No	757 (61.2)	296 (46.5)	461 (76.8)	
Alcohol/substance use disorder (comorbid)^d^				
Yes	125 (10.1)	89 (14.0)	36 (6.0)	<.001
No	1111 (89.9)	547 (86.0)	564 (94.0)	

Abbreviation: NA, not applicable.

^a^ Ethnicity was self-reported.

^b^ Includes Arab, Bangladeshi, Chinese, Indian, mixed race, Pakistani, other Asian, and other.

^c^ *International Statistical Classification of Diseases and Related Health Problems, Tenth Revision* code.

^d^ Excluding cannabis use disorder.

The primary outcome measure was the proportion of cases transferred from a general ward to PICU on days 3 to 5 after presentation to hospital. A total of 31.0% of current users and 24.2% of nonusers (OR, 1.41; 95% CI, 1.10-1.81; *P* = .008; aOR, 1.36; 95% CI, 1.01-1.81; *P* = .04; Table 2) were transferred during this period (Figure). Post hoc analyses revealed that the risk of transfer was elevated in women (aOR, 2.03; 95% CI, 1.22-3.39; *P* = .007) and those older than 35 years (aOR, 2.53; 95% CI, 1.52-4.21; *P* < .001) (Figure; eFigure 3 in Supplement 1). Post hoc analyses found that cannabis users were less likely to be transferred on days 6 to 8 and days 7 to 9 (eTable 3 in Supplement 1).

**Table 2.** Univariate and Multivariable Analysis of the Risk Factors for Transfer to Psychiatric Intensive Care Unit on Days 3 to 5 Postpresentation

Characteristic	Univariate		Multivariable	
	Odds ratio (95% CI)	*P* value	Odds ratio (95% CI)	*P* value
Cannabis use				
Past/never user				
Current user	1.41 (1.10-1.81)	.008	1.36 (1.01-1.81)	.04
Age	0.99 (0.97-1.00)	.02	0.99 (0.98-1.00)	.12
Gender				
Male	1 [Reference]	1 [Reference]	1 [Reference]	1 [Reference]
Female	1.12 (0.86-1.45)	.41	1.21 (0.91-1.60)	.19
Ethnicity^a^				
Black	1 [Reference]	1 [Reference]	1 [Reference]	1 [Reference]
White	0.83 (0.61-1.12)	.22	0.84 (0.60-1.17)	.29
Other^b^	1.20 (0.85-1.71)	.30	1.16 (0.81-1.66)	.41
Primary diagnosis				
Substance use disorder (F1x^c^)	1.78 (1.01-3.16)	.05	1.55 (0.83-2.90)	.17
Psychotic disorder (F2x^c^)	1 [Reference]	1 [Reference]	1 [Reference]	1 [Reference]
Affective disorder (F3x^c^)	1.10 (0.82-1.46)	.53	1.18 (0.88-1.59)	.27
Other	1.16 (0.77-1.75)	.49	1.17 (0.76-1.80)	.48
Tobacco use				
Yes	1.15 (0.87-1.50)	.33	1.12 (0.83-1.51)	.45
No	1 [Reference]	1 [Reference]	1 [Reference]	1 [Reference]
Stimulant use				
Yes	1.04 (0.81-1.35)	.75	0.91 (0.69-1.21)	.53
No	1 [Reference]	1 [Reference]	1 [Reference]	1 [Reference]
Alcohol/substance use disorder (comorbid)^d^				
Yes	1.31 (0.88-1.95)	.18	1.20 (0.76-1.89)	.44
No	1 [Reference]	1 [Reference]	1 [Reference]	1 [Reference]
Admission year	1.02 (0.99-1.05)	.28	1.01 (0.98-1.05)	.42

^a^ Ethnicity was self-reported.

^b^ Includes Arab, Bangladeshi, Chinese, Indian, mixed race, Pakistani, other Asian, and other.

^c^ *International Statistical Classification of Diseases and Related Health Problems, Tenth Revision *code.

^d^ Excluding cannabis use disorder.

**Figure.**
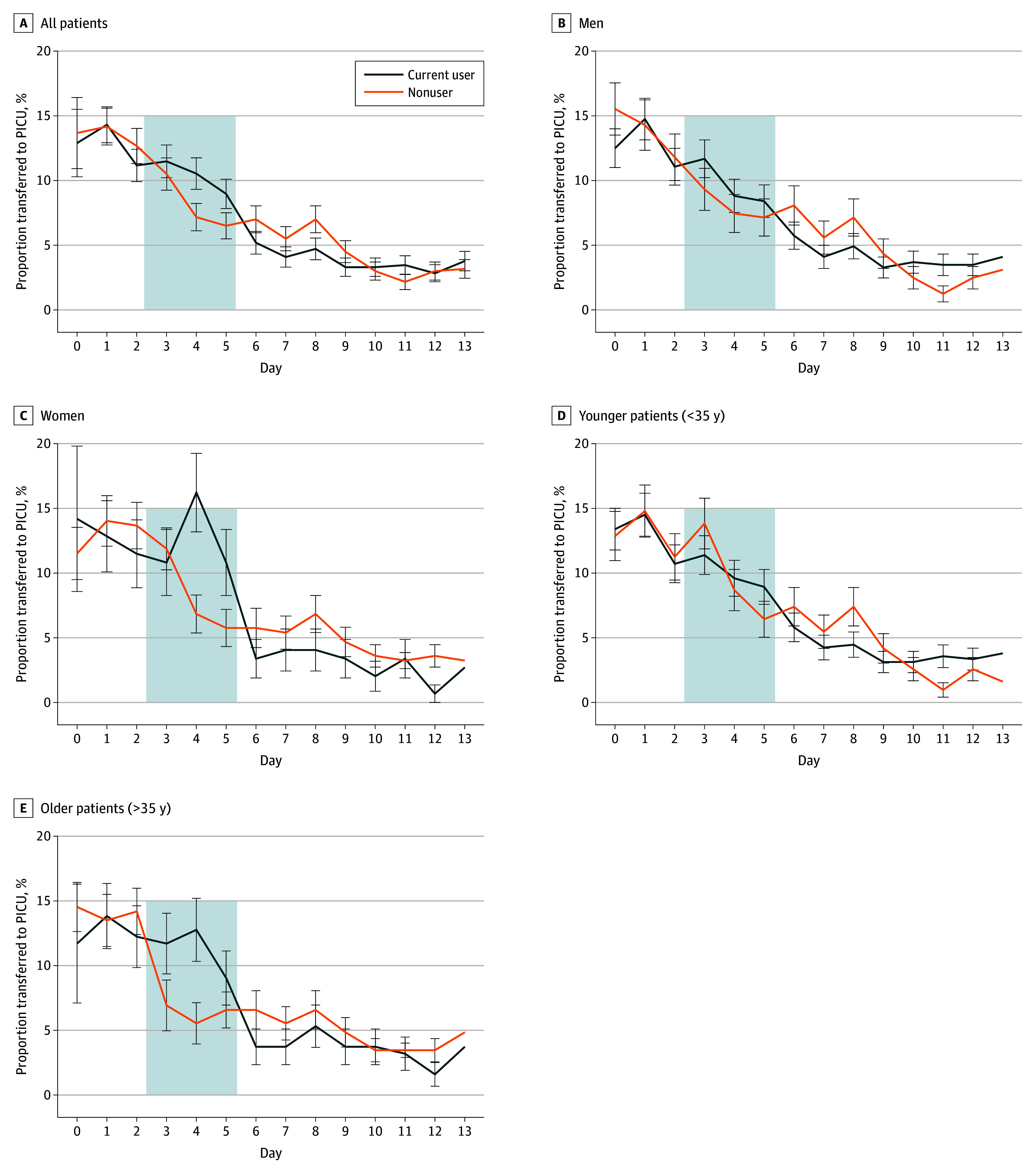
Days From Presentation to Hospital Until Transfer to Psychiatric Intensive Care Unit (PICU) Data presented are the proportion of each group who are transferred to PICU on each day after admission. Error bars are standard error.

## Discussion

This study found that patients who had been using cannabis prior to psychiatric hospital admission were more likely to need intensive clinical care. In particular, it showed that cannabis users were more likely than nonusers to be transferred to PICU 3 to 5 days after presentation to hospital, the period when cannabis withdrawal is most evident. These findings build upon previous research that described episodes of psychosis which appear to have been triggered by CWS.^10^

In post hoc analyses, we found that the associations were driven by women and older patients. The endocannabinoid system is sexually dimorphic and there is evidence that women experience more severe cannabis withdrawal.^11^ There is also some evidence from psychosis populations that those with a severe cannabis use disorder at illness onset are more likely to continue to use drugs, an association that could explain the association observed in older patients.^12^

In a meta-analysis, 40% of regular cannabis users from inpatient samples reported CWS.^13^ In one study, 47% of participants reported at least 4 symptoms that were of a severe intensity.^5^ A systematic review of studies investigating cannabinoid agonist replacement therapy for CWS included 10 double-blind placebo-controlled clinical trials and found promising effects on withdrawal symptoms and treatment retention, as well as encouraging safety data.^14^ However, none of the trials recruited patients with severe psychiatric disorders. This is a important limitation, as the risks associated with cannabinoid receptor agonists may be greater in people with a psychiatric disorder.^2^

### Strengths and Limitations

The strengths of this study include the large sample size and use of a rich clinical dataset. The main limitation of the study is the quality of data on cannabis use, which relied on clinical assessments that may not be accurate.^15^ We did not assess cannabis withdrawal symptoms, the extent of cannabis use, the severity of cannabis use disorder, or equivalent metrics for tobacco and stimulant use. Additionally, we did not collect data on the prescription of antipsychotic medications, benzodiazepines, or nicotine replacement therapy, which could potentially mitigate the effects of cannabis withdrawal.^16^

## Conclusions

In summary, this study found that cannabis use is associated with an increased risk of transfer to PICU between 3 and 5 days after presentation to hospital—a period associated with the peak severity of CWS. Further research is needed to explore the safety and efficacy of treatments for CWS in psychiatric populations.

## Notes

Supplement 1. eFigure 1. Study flowchart eMethods. Assessment of cannabis use: agreement between manual review and natural language processing application eTable 1. Demographic and clinical characteristics of the entire study population (N=52,088) eTable 2. Multivariable analysis of the risk factors for admission and transfer to PICU at any timepoint (N=52,088) eFigure 2. Time profile of transfers to PICU in past cannabis users and never/minimal users eTable 3. Effect of cannabis use on risk of transfer to PICU for each 3-day period after presentation to hospital eMethods 2. Subgroup analyses of age and gender eFigure 3. Subgroup analysis

Supplement 2. Data sharing statement
