# Supplementary material for: Cannabis Withdrawal and Psychiatric Intensive Care
Source: JAMA Psychiatry. 2025 Jun 11;82(8):838–43. doi: 10.1001/jamapsychiatry.2025.1216 (PMC12159852; doi:10.1001/jamapsychiatry.2025.1216)
Supplement: Supplement 1. — eFigure 1. Study flowchart eMethods. Assessment of cannabis use: agreement between manual review and natural language processing application eTable 1. Demographic and clinical characteristics of the entire study population (N=52,088) eTable 2. Multivariable analysis of the risk factors for admission and transfer to PICU at any timepoint (N=52,088) eFigure 2. Time profile of transfers to PICU in past cannabis users and never/minimal users eTable 3. Effect of cannabis use on risk of transfer to PICU for each 3-day period after presentation to hospital eMethods 2. Subgroup analyses of age and gender eFigure 3. Subgroup analysis [file jamapsychiatry-e251216-s001.pdf]

Supplemental Online Content

Malik A, Shetty H, Oliver D, et al. Cannabis withdrawal and psychiatric intensive care. *JAMA Psychiatry*. Published online June 11, 2025. doi:10.1001/jamapsychiatry.2025.1216

eFigure 1. Study flowchart
eMethods. Assessment of cannabis use: agreement between manual review and natural language processing application
eTable 1. Demographic and clinical characteristics of the entire study population (N=52,088)
eTable 2. Multivariable analysis of the risk factors for admission and transfer to PICU at any timepoint (N=52,088)
eFigure 2. Time profile of transfers to PICU in past cannabis users and never/minimal users
eTable 3. Effect of cannabis use on risk of transfer to PICU for each 3-day period after presentation to hospital
eMethods 2. Subgroup analyses of age and gender
eFigure 3. Subgroup analysis

This supplemental material has been provided by the authors to give readers additional information about their work.

© 2025 Malik A et al. *JAMA Psychiatry*.

eFigure 1: Study flowchart

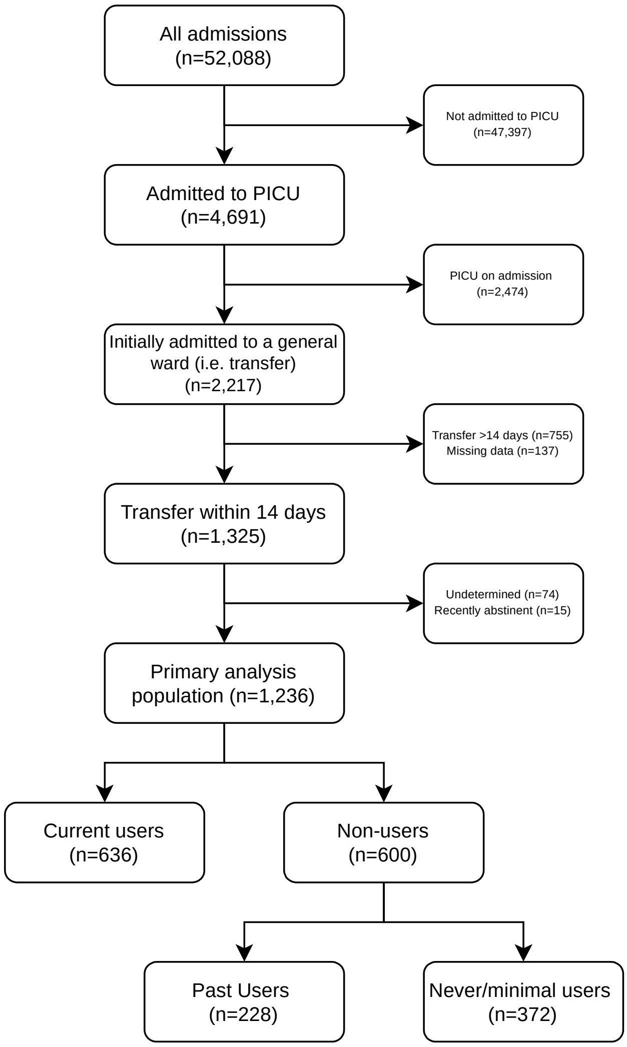

© 2025 Malik A et al. *JAMA Psychiatry*.

eMethods 1. Assessment of cannabis use: agreement between manual review and natural language processing

For manual review alone, the inter-rater reliability for ‘current user’ vs. ‘non-user’ status across 50 randomly selected participants was 0.88 (Cohen’s kappa).

Comparing manual review and natural language processing, the overall agreement was 79% (Cohen's kappa = 0.57). Of the 634 current users identified through manual review, 614 (97%) were determined to be current cannabis users by NLP. Of the 372 never/minimal users, 301 (81%) were identified as non-users by NLP. For past users (n=228) there was more disagreement: NLP identified 57 (25%) as non-users and 171 (75%) as users.

© 2025 Malik A et al. *JAMA Psychiatry*.

eTable 1. Demographic and clinical characteristics of the entire study population (N=52,088)					
		All patients	Current users	Non-users	P value
	n	52,088 (100.0%)	24,579 (47.2%)	27,509 (52.8%)	
	Age (years, SD)	41.8 (15.255)	36.8 (12.072)	46.3 (16.368)	<0.001
Gender	Female	23,091 (44.3%)	7,935 (32.3%)	15,156 (55.1%)	
	Male	28,991 (55.7%)	16,640 (67.7%)	12,351 (44.9%)	<0.001
	Not known	6 (0.0%)	4 (0.0%)	2 (0.0%)	
Ethnicity (self-reported)	Black	18,422 (35.4%)	9,662 (39.3%)	8,760 (31.8%)	
	White	25,721 (49.4%)	11,273 (45.9%)	14,448 (52.5%)	
	Other	7,945 (15.3%)	3,644 (14.8%)	4,301 (15.6%)	<0.001
Primary diagnosis	Substance use disorder (F1x)	7,688 (14.8%)	4,535 (18.5%)	3,153 (11.5%)	
	Psychotic disorder (F2x)	21,247 (40.8%)	10,924 (44.4%)	10,323 (37.5%)	
	Affective disorder (F2x)	10,542 (20.2%)	4,071 (16.6%)	6,471 (23.5%)	
	Other	12,611 (24.2%)	5,049 (20.5%)	7,562 (27.5%)	<0.001
Cannabis use	Yes	24,579 (47.2%)	24,579 (100.0%)	-	
	No	27,509 (52.8%)	-	27,509 (100.0%)	n/a
Tobacco use	Yes	28,664 (55.0%)	17,607 (71.6%)	11,057 (40.2%)	
	No	23,424 (45.0%)	6,972 (28.4%)	16,452 (59.8%)	<0.001
Stimulant use	Yes	15,774 (30.3%)	12,342 (50.2%)	3,432 (12.5%)	
	No	36,314 (69.7%)	12,237 (49.8%)	24,077 (87.5%)	<0.001
Substance use disorder (co-morbid)*	Yes	11,247 (21.6%)	6,577 (26.8%)	4,670 (17.0%)	
	No	40,841 (78.4%)	18,002 (73.2%)	22,839 (83.0%)	<0.001
*excluding cannabis use disorder					

© 2025 Malik A et al. *JAMA Psychiatry*.

eTable 2. Multivariable analysis of the risk factors for admission and transfer to PICU at any timepoint (N=52,088)							
		Admission (including transfers)			Transfer		
		Odds ratio	95% CI	P value	Odds ratio	95% CI	P value
Cannabis use	Past/never user	-	-	-	-	-	-
	Current user	1.44	(1.33-1.55)	<0.0001	1.37	(1.23-1.53)	<0.0001
Age		0.97	(0.96-0.97)	<0.0001	0.96	(0.95-0.96)	<0.0001
Gender	Male	-	-	-	-	-	-
	Female	0.51	(0.48-0.55)	<0.0001	0.78	(0.71-0.86)	<0.0001
Ethnicity (self-reported)	Black	-	-	-	-	-	-
	White	0.38	(0.35-0.41)	<0.0001	0.47	(0.42-0.53)	<0.0001
	Other	0.50	(0.46-0.55)	<0.0001	0.60	(0.53-0.68)	<0.0001
Primary diagnosis	Substance use disorder (F1x)	0.26	(0.22-0.31)	<0.0001	0.24	(0.19-0.32)	<0.0001
	Psychotic disorder (F2x)	-	-	-	-	-	-
	Affective disorder (F3x)	1.17	(1.08-1.27)	0.0001	1.20	(1.08-1.34)	0.0009
	Other	0.38	(0.35-0.42)	<0.0001	0.33	(0.28-0.38)	<0.0001
Tobacco use	Yes	1.37	(1.27-1.47)	<0.0001	1.32	(1.19-1.45)	<0.0001
	No	-	-	-	-	-	-
Stimulant use	Yes	1.41	(1.31-1.52)	<0.0001	1.35	(1.22-1.50)	<0.0001
	No	-	-	-	-	-	-
Substance use disorder (co-morbid)*	Yes	0.80	(0.71-0.90)	0.0002	0.78	(0.66-0.93)	0.0044
	No	-	-	-	-	-	-
Admission year		1.03	(1.02-1.04)	<0.0001	1.02	(1.01-1.03)	0.0011
*excluding cannabis use disorder							

© 2025 Malik A et al. *JAMA Psychiatry*.

eFigure 2: Time profile of transfers to PICU in past cannabis users and never/minimal users

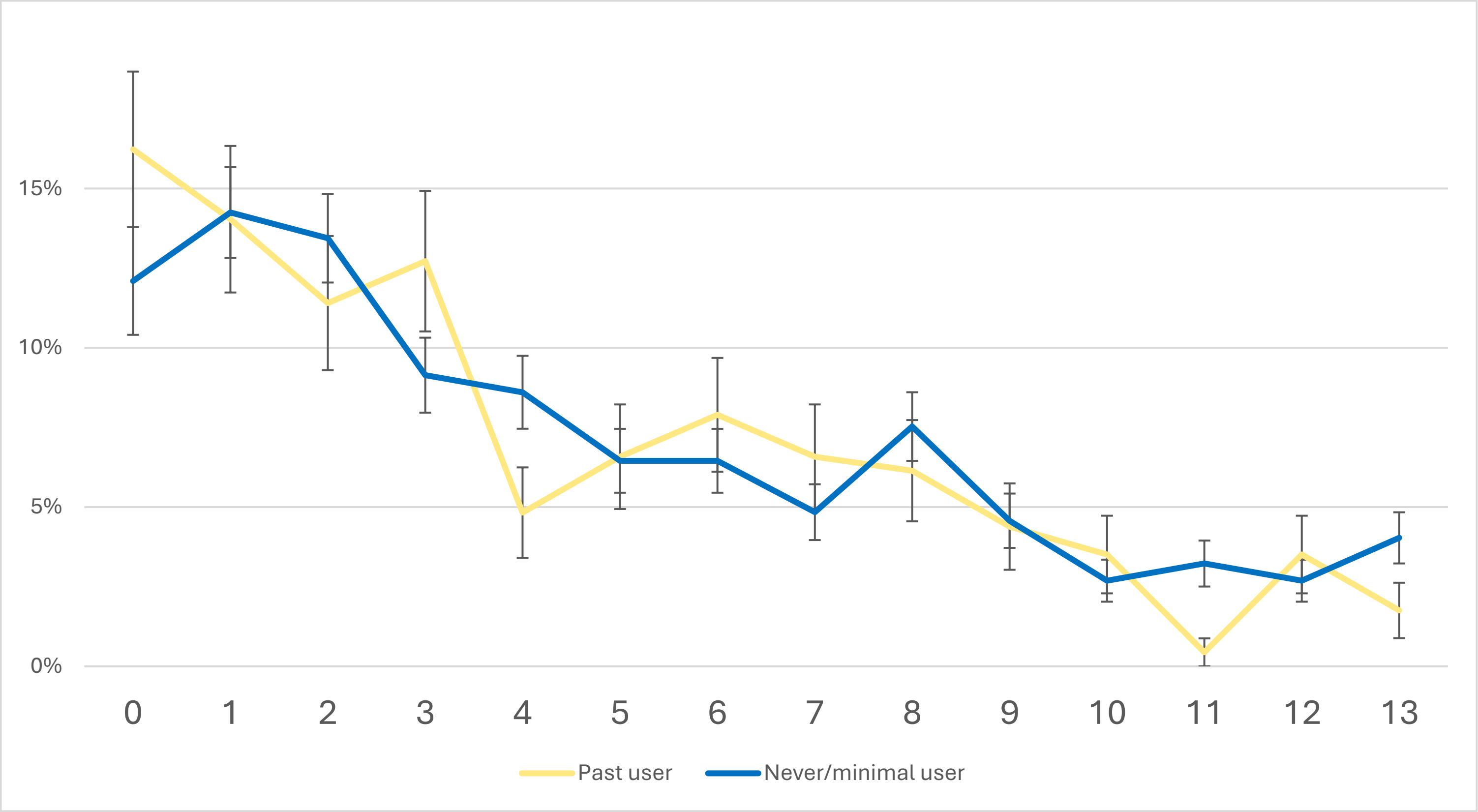

Days from presentation to hospital until transfer to PICU. Data presented are the proportion of each group who are transferred to PICU on each day after presentation. Error bars are Standard Error.
Yellow: 'Past users'
Blue: 'Never/minimal users'

© 2025 Malik A et al. *JAMA Psychiatry*.

eTable 3. Effect of current cannabis use on risk of transfer to PICU for each 3-day period after presentation to hospital		
Period	Adjusted Odds Ratio (95% CI)	P value
0-2	1.04 (0.80 to 1.36)	0.76
1-3	1.01 (0.77 to 1.32)	0.94
2-4	1.14 (0.87 to 1.51)	0.34
3-5	1.36 (1.01 to 1.81)	0.04
4-6	1.21 (0.89 to 1.65)	0.22
5-7	0.82 (0.59 to 1.14)	0.24
6-8	0.54 (0.38 to 0.77)	0.0006
7-9	0.59 (0.41 to 0.86)	0.006
8-10	0.74 (0.51 to 1.09)	0.13
9-11	1.17 (0.76 to 1.81)	0.48
10-12	1.29 (0.81 to 2.04)	0.28
11-13	1.23 (0.79 to 1.94)	0.36

© 2025 Malik A et al. *JAMA Psychiatry*.

eMethods 2. Subgroup analyses of age and gender

As age and gender appeared to have a moderating effect in the multivariable analysis ($p<0.2$), we completed post-hoc analyses of these subgroups (Figure 1 & eFigure3).

For females, the odds of transfer during the risk period were increased (adjusted OR=2.03 [95% CI: 1.22-3.39], p=0.007), but the association was not statistically significant in males (adjusted OR=1.10 [95% CI: 0.77-1.57], p=0.60).

For patients over the age of 35, the odds of transfer during the risk period were increased (adjusted OR=2.53 [95% CI: 1.52-4.21], p=0.0004), but there was no association in those <35 years (adjusted OR=0.98 [95% CI: 0.69-1.40], p=0.91).

Analyses stratified by both age and gender are presented in eFigure 3, the largest effect was observed in women over the age of 35 (adjusted OR = 4.28 [95% CI: 1.73-10.6], p=0.002). The association was also statistically significant in males over the age of 35 (adjusted OR = 1.93 [95% CI: 1.02-3.68], p=0.045).

eFigure 3. Risk of transfer to PICU during the cannabis withdrawal risk period: subgroup analysis

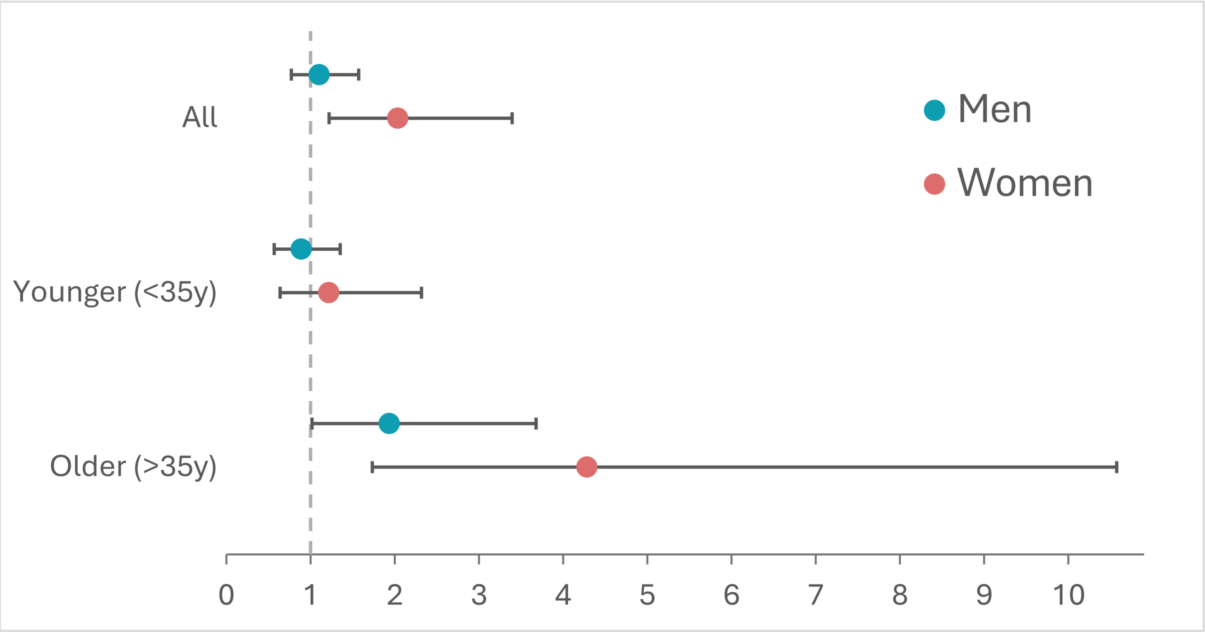

Forest plot reporting the effect of current cannabis use on the odds of transfer to PICU during the cannabis withdrawal risk period (days 3-5) in subgroups split by age and gender. Data presented are adjusted Odds Ratios from multivariable analyses. Error bars are 95% confidence intervals.

© 2025 Malik A et al. *JAMA Psychiatry*.
