# Supplementary material for: Cannabis Withdrawal and Psychiatric Intensive Care
Source: JAMA Psychiatry. 2025 Jun 11;82(8):838–43. doi: 10.1001/jamapsychiatry.2025.1216 (PMC12159852; doi:10.1001/jamapsychiatry.2025.1216)
Supplement: Supplement 2. — Data sharing statement [file jamapsychiatry-e251216-s002.pdf]

**Data Sharing Statement**

**Data**
**Data available:** No

**Additional Information**
**Explanation for why data not available:** Data are owned by a third party, Maudsley Biomedical Research Centre (BRC) Clinical Records Interactive Search tool, which provides access to anonymised data derived from SLaM electronic medical records. These data can only be accessed by permitted individuals from within a secure firewall (i.e. the data cannot be sent elsewhere), in the same manner as the authors. For more information please contact: cris.administrator@slam.nhs.uk.
